# Neural activation associated with outgroup helping in adolescent rats

**DOI:** 10.1016/j.isci.2022.104412

**Published:** 2022-05-16

**Authors:** Jocelyn M. Breton, Jordan S. Eisner, Vaidehi S. Gandhi, Natalie Musick, Aileen Zhang, Kimberly L.P. Long, Olga S. Perloff, Kelsey Y. Hu, Chau M. Pham, Pooja Lalchandani, Matthew K. Barraza, Ben Kantor, Daniela Kaufer, Inbal Ben-Ami Bartal

**Affiliations:** 1 Helen Wills Neuroscience Institute, University of California Berkeley, Berkeley, CA 94720, USA; 2 Molecular and Cellular Biology, University of California Berkeley, Berkeley, CA 94720, USA; 3 Integrative Biology, University of California Berkeley, Berkeley, CA 94720, USA; 4 Canadian Institute for Advanced Research, Toronto, ON M5G1M1, Canada; 5 School of Psychological Sciences and Sagol School of Neuroscience, Tel-Aviv University, Tel Aviv-Yafo, Israel, 6997801

**Keywords:** Behavioral neuroscience, Cellular neuroscience

## Abstract

Prosocial behavior, helping others in need in particular, occurs preferentially in response to the perceived distress of one’s own group members or ingroup. To investigate the development of ingroup bias, neural activity during a helping test was analyzed in adolescent and adult rats. Although adults selectively released trapped ingroup members, adolescent rats helped both ingroup and outgroup members, suggesting that ingroup bias emerges in adulthood. Analysis of brain-wide neural activity, indexed by expression of the early-immediate gene c-Fos, revealed increased activity for ingroup members across a broad set of regions previously associated with empathy. Adolescents showed reduced hippocampal and insular activity and increased orbitofrontal cortex activity compared to adults. Non-helper adolescents demonstrated increased amygdala connectivity. These findings demonstrate that biases for group-dependent prosocial behavior develop with age in rats and suggest that specific brain regions contribute to prosocial selectivity, pointing to possible targets for the functional modulation of ingroup bias.

## Highlights

• Adolescents rats — but not adult rats — helped outgroup members, releasing them from a trap
• Neural activity was higher for ingroup members across some regions independent of age
• Activity was modulated by age in the insula, hippocampus, hypothalamus, and vOFC
• Adolescent “non-openers” showed increased amygdala connectivity

## Graphical abstract

**Figure:**
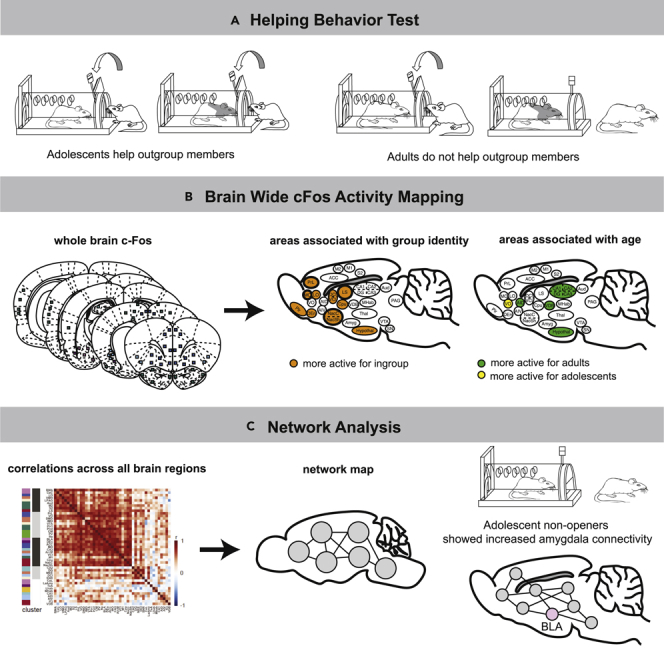

## Introduction

Responding to another’s distress with a prosocial action is a crucial component of life in social groups (Van and Burkart, 2011; Wilson, 2012; Dugatkin, 1997). Prosocial actions are any that occur with the intention of benefiting others or improving their well-being (Cronin, 2012). Distress is a salient signal that can elicit empathy in the observer and recruit motivational responses intended on helping the distressed individual (Decety et al., 2016). Here, empathy is defined as the ability to perceive and share the emotions of others, coupled with a motivation to care for their well-being (Decety et al., 2016; Batson, 2009). Yet, in humans and other species, the empathic response to distressed others is largely impacted by their social identity. Congruently, prosocial behavior is selective and preferentially extended to affiliated others (Hamilton, 1964; Hackel et al., 2017). To put it simply, we are much more likely to help others we care about than those we do not. For humans and other social species, affiliation expands beyond individual familiarity to encompass others of the same social group or ingroup members (Bernhard et al., 2006; Kavaliers and Choleris, 2017). Identifying others as ingroup or outgroup members thus comprises a quick, effective heuristic for determining prosocial motivation. This mechanism, ostensibly adaptive at its origin, can be detrimental in modern society, leading to prejudice, discrimination, and negative stereotyping. For example, a lack of empathy for outgroup members gives rise to intergroup conflict, which is a ubiquitous phenomenon throughout human history and has devastating effects on society at large, as well as at the individual level as it negatively impacts mental and physical wellbeing (Cranna, 1994; Fiske, 2002; Olson, 2014). Yet, social bias, in particular with regard to prosocial motivation, is difficult to influence (Fiske, 2002). Targeting the formation of social bias during development, when social information is especially salient (Andrews et al., 2021; Kilford et al., 2016) yet flexible (Fuhrmann et al., 2015), is a promising strategy for influencing behavior toward outgroup members along the life span, and understanding the neural mechanisms underlying the development of social bias is thus a key question of our time.

Evidence suggests that children categorize others into ingroup and outgroup members and demonstrate social preferences very early in development (Decety et al., 2021; Charlesworth and Banaji, 2021). For instance, babies prefer faces of same-race adults (Xiao et al., 2018) or adults with the same accent as their caretakers (Kinzler et al., 2007) and use group membership information to guide behavioral choices (Shutts et al., 2010, 2009). By 3–4 years of age, children can show ingroup favoritism (Aboud, 2003; Hailey and Olson., 2013), even toward arbitrarily determined groups (Bigler et al., 1997). However, distress is a unique signal, and children are highly sensitive to others’ well-being. At 9 months of age, children prefer prosocial actions over harmful ones; by the end of their first year, they begin to comfort others; and by their second year of life, they engage in helping behavior (Gross et al., 2015; Svetlova et al., 2010; Decety et al., 2021). Thus, although social identity influences social motivation in children including increased loyalty, sharing, and positive attitudes toward ingroup members (Over, 2018; Bigler et al., 1997), it is unclear whether empathic helping is similarly prone to ingroup bias at young ages. Furthermore, encouragingly, children are more malleable than adults in their biases toward outgroup members (Nelson et al., 2016). Several studies have found that in humans, exposure to a diverse environment during childhood is associated with reduced biases into adulthood (Telzer et al., 2013, Zuo and Han, 2013, Heron-Delaney et al., 2011; Anzures et al., 2012). For example, unlike infants raised by families of their own race, infants in a multiracial community do not prefer same-race faces (Bar-Haim et al., 2006). Ingroup vs. outgroup categorization is thus flexible during human development. Yet, critically, the neural basis of the development of prosocial biases remains undefined and could provide key insights into the flexibility of this biological mechanism.

Animal models have proved useful in the study of neural circuits underlying prosocial behavior. During the helping behavior test (HBT), adult rats that are exposed to a distressed trapped rat are motivated by that distress (Ben-Ami Bartal et al., 2016) to open a restrainer and release the trapped rat, even in the lack of social contact (Ben-Ami Bartal et al., 2011), demonstrating empathic helping. However, this prosocial behavior is socially selective, as rats release others from their own genetic strain but do not help rats from unfamiliar strains (Ben-Ami Bartal et al., 2021; Ben-Ami Bartal et al., 2014). Furthermore, two weeks of cohabitation with a member of an unfamiliar strain causes a prosocial shift toward strangers of that strain, indicating that for rats, group membership is flexibly determined by social experience (Ben-Ami Bartal et al., 2021; Ben-Ami Bartal et al., 2014). The HBT is thus a good model for studying the neural processes involved in social bias for empathic helping in rats. Indeed, we found that neural regions typically associated with empathy, as well as reward, were active in rats following the HBT with trapped ingroup members (Ben-Ami Bartal et al., 2021). In contrast, rats tested with outgroup members only showed activity in empathy networks. This pattern was not observed for non-trapped others, or for a nonsocial reward. Thus, although rats typically activate regions associated with negative arousal during the HBT, activity in the reward & motivation system is selectively associated with the presence of ingroup members and is predictive of helping.

Although we have studied the neural bases of prosocial biases in adult animals, they have not yet been explored within a developmental context. Here, we turned to young rats to examine the way adolescent brains respond to ingroup and outgroup members in distress during the HBT. We found that adolescent rats consistently released trapped outgroup members, in stark contrast to adults. Distinct patterns of movement and social interactions for ingroup and outgroup members suggest adolescents distinguish between the two conditions. After a final HBT session, a neural activity marker, the immediate early gene c-Fos, was analyzed to identify the neural networks associated with prosocial behavior. Distinct patterns of neural activity associated with each condition were observed and may underlie the generalized helping observed in adolescents compared to adults. In general, adolescents activated similar regions as adults during the HBT, reinforcing the participation of empathy and reward regions in this task. However, the hippocampus of adolescents was less active than adults, whereas activity in the orbitofrontal cortex was elevated. These findings suggest that the response to distress in adults may be inhibited by circuits that respond to social category information, including hippocampal circuits (Hitti and Siegelbaum, 2014). Overall, our findings demonstrate that adolescent rats do not show a similar ingroup bias as adults and display altered activity in networks of social mapping and reward valuation.

## Results

### Unlike adult rats, adolescent rats do not demonstrate an ingroup bias for prosocial behavior

**Figure 1:**
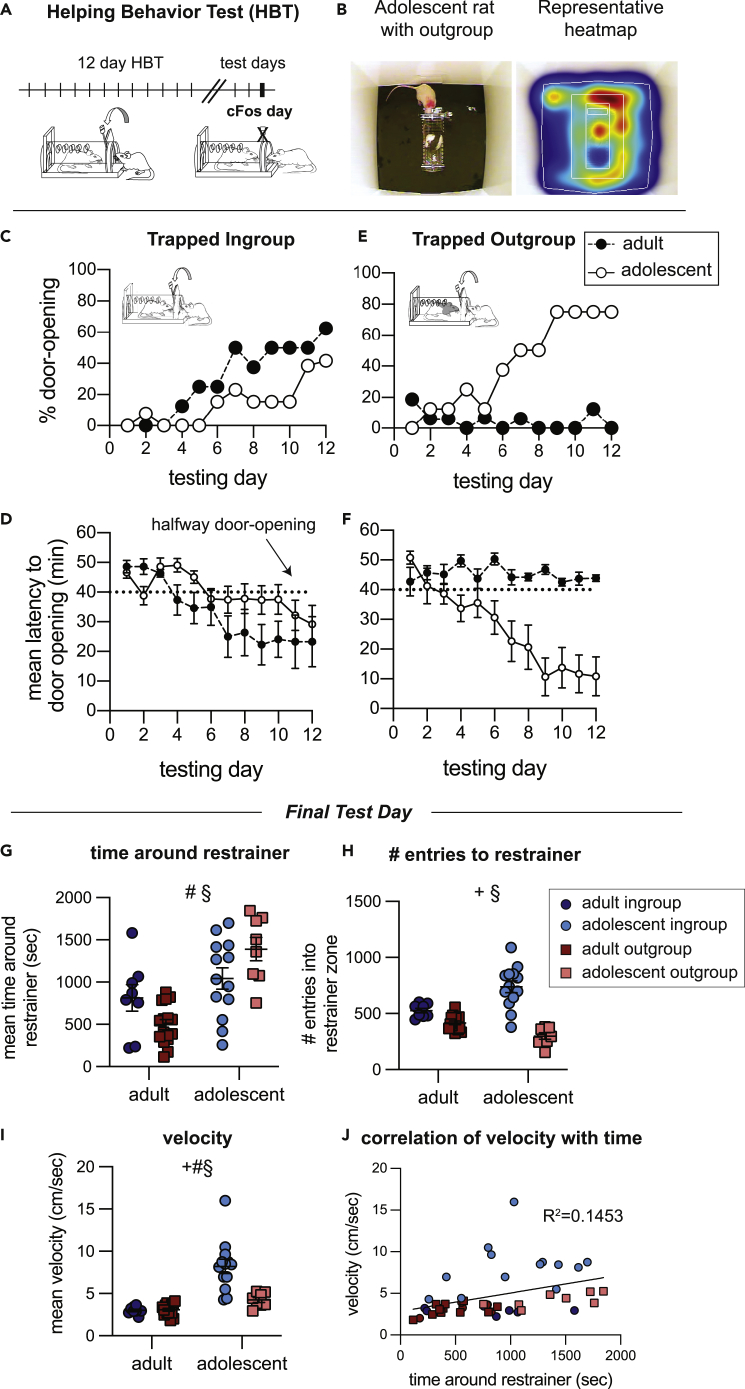
Helping behavior for adult and adolescent rats Unlike adults, adolescent rats do not demonstrate an ingroup bias for prosocial behavior. (A) Diagram of the helping behavior test. (B) Representative movement pattern of an adolescent tested with an outgroup member depicted by a heatmap of the rat’s location along the session. (C–F) Helping behavior is expressed by % of door-openings and latency to open for the ingroup (C–D) and outgroup (E–F) across testing sessions. The dashed line indicates the half-way door-opening by the experimenter. (G–J) Analysis of movement patterns in the final testing session, including: (G) The time rats spent near the trapped rat, (H) the number of entries into the zone around the restrainer and (I) average velocity. Error bars represent S.E.M. (J) Time around the restrainer was correlated with activity levels. 2-way ANOVA: + main effect of group identity, # main effect of age, § significant interaction between age and group identity.

Rats were tested in the helping behavior test (HBT), a simple test where rats can learn to open the door to a restrainer and release a conspecific trapped inside, as previously described in (Ben-Ami Bartal et al., 2011). For hourly daily sessions over a two-week period, rats were given the opportunity to open the restrainer, and they were not trained beforehand or rewarded in any way other than the reward afforded by door-opening and any subsequent social interaction. Here, helping behavior was studied in adolescent Sprague-Dawley (SD) rats (p32 days old) tested with age-matched SD cagemates (‘adolescent ingroup’, n = 13) or with age-matched rats of the unfamiliar black-caped Long-Evans (LE) strain (‘adolescent outgroup’ n = 8 Figures 1A and 1B). Adolescent helping was compared to adult rats (p60-p90) tested with the same protocol (‘adult ingroup’, n = 8 & ‘adult outgroup’, n = 16). Part of this dataset was previously published in (Ben-Ami Bartal et al., 2021).

Like adults, adolescent rats tested with ingroup members were motivated to release their trapped cagemates as expressed by a significant increase in percent door-openings (Cochrans’ Q, p < 0.01) and reduced latency to door-opening (Friedman, p < 0.05) along the days of testing (Figures 1C and 1D, Video S1). Strikingly, unlike adults, adolescent rats robustly released trapped outgroup members as expressed by a significant increase in the percent of door-openings (Cochrans’ Q, p < 0.001) and decreased latency to open the restrainer door (Friedman, p < 0.01, Figures 1E and 1F, Video S2). Nearly all rats in this condition (n = 6/8) consistently released the trapped outgroup member as opposed to 0/16 in the adult condition. The percent of door-openings did not increase in the adult outgroup condition and door-opening behavior was rarely observed (Cochrans’ Q, Friedman, p > 0.05).

Video S1. Adult and adolescent rats help ingroup members. Related to Figure 1

Video S2. Adolescents but not adults help outgroup members. Related to Figure 1

This unexpected finding demonstrates that the lack of prosocial motivation toward outgroup members emerges after early adolescence or in adulthood.

### Adolescent rats interact differently with ingroup and outgroup members

An unexpected finding was that adolescent rats were less successful at helping trapped ingroup members compared to adults. Only 4/13 adolescent rats became consistent openers by the end of testing compared to 6/8 adult rats. This could point to reduced motivation to release trapped cagemates. Yet, movement data was similar to that observed in the groups who consistently opened (the adult-ingroup and adolescent outgroup), and these rats showed high neural activation in the regions previously associated with helping (Ben-Ami Bartal et al., 2021) (described in detail below), which may suggest otherwise. On the final testing day, on which neural activity was later indexed, the restrainer was latched so that all rats had an objectively similar experience of being in the presence of a trapped conspecific for the entire session length. On this final test day, adolescents in the ingroup condition spent a similar amount of time around the trapped rat as the adolescent outgroup rats; yet, they entered the zone around the restrainer more frequently and were more active than the outgroup condition (ANOVA, p < 0.01, Bonferroni p < 0.01, Figures 1G–1I, Video S3). Thus, despite lower rates of door-opening for adolescent ingroup than outgroup members, adolescents in the ingroup condition demonstrated movement patterns that reflect increased interest in the trapped rat, which may indicate motivation to release the trapped cagemate. In general, as is typically observed, adolescents in both conditions were more active than adults (Figure 1I). They also spent more time near the trapped rat than did adults on the final session (ANOVA, p < 0.01, Bonferroni p < 0.01, Figures 1G and 1H), suggesting that a social stimulus is more salient for adolescents. Across all groups, activity was directed at the trapped rat; there was a positive correlation between activity and time near the restrainer (Pearson’s, p < 0.01, Figure 1J), and rats were observed circling the restrainer as demonstrated in Figure 1B and Video S3. Combined, these data suggest that adolescents tested with cagemates were motivated but less successful at learning the door-opening task than the adolescents tested with outgroup members. Future studies will be needed to explore the possible processes involved in this finding.

Video S3. Activity patterns on the final day of testing. Related to Figure 1

**Figure 2:**
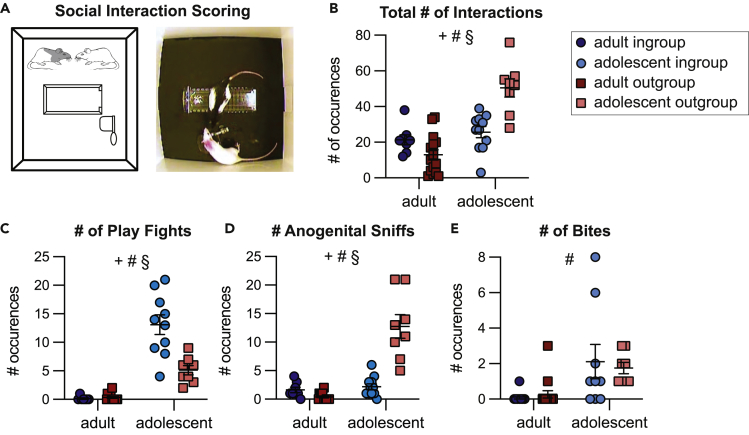
Social behavior for adults and adolescent rats upon door opening Adolescent rats display different types of social interaction depending on group identity. (A) Diagram and representative image of social interaction between an adolescent SD and LE rat. (B) Compared to adults, adolescents had a higher number of total social interactions scored within the 5-min period. This includes all types of interactions, including play fighting, touching, and investigations. (C) The number of play fights was highest in adolescents tested with cagemates (D) The number of investigative anogenital sniffs was highest in adolescents tested with strangers. (E) The number of bites did _not_ differ between adolescent groups. 2-way ANOVA: + main effect of group identity, # main effect of age, § significant interaction between age and group identity. Error bars represent S.E.M.

To further explore the motivational state of adolescents with trapped ingroup and outgroup members, social interactions immediately after door-opening were quantified on the day before the last session (the final day where social interaction was afforded, Figure 2A, see STAR methods). In line with the movement data, adolescents interacted with the freed conspecific more than adults (ANOVA, main effect of age, p < 0.05), reinforcing the increased salience of social interaction for adolescents. Adolescents in the outgroup condition also showed the greatest number of interactions (Bonferroni, p < 0.001, Figure 2B). Yet, the type of interaction was markedly different for adolescent ingroup and outgroup pairs; play fighting emerged as the predominant interaction in the adolescent ingroup condition (Bonferroni p < 0.001, Figure 2C), whereas play was rarely observed with adolescent outgroup members, but nonplay interactions, including anogenital sniffs, were significantly higher (Bonferroni p < 0.001, Figure 2D). Aggressive behaviors such as biting were rarely seen in any group and did not differ across the adolescent conditions (Figure 2E). Thus, even on the final days of testing, rats behaved differently with ingroup and outgroup members, indicating they could distinguish between these social identities.

Altogether, we take these data to indicate that adolescents were more motivated than adults to release and interact with the trapped rat. The differing behaviors between the adolescent ingroup and outgroup conditions suggest that the free rats were sensitive to the group identity of the trapped rat and may point to different motivational states in these conditions. For example, although we cannot tease apart if prosocial behavior was because of an empathic response and/or because of a desire for social interaction, rats in the adolescent ingroup condition may be more motivated by play whereas rats in adolescent outgroup condition may be more motivated by a desire for social investigation. Importantly, even if future work finds that rats of all ages experience less emotional contagion with outgroup members in the HBT, here, adolescents, in contrast with adults, release the trapped rat, demonstrating prosocial motivation and lack of social bias.

### Neural activity patterns in the helping behavior test correspond with age and group membership

**Figure 3:**
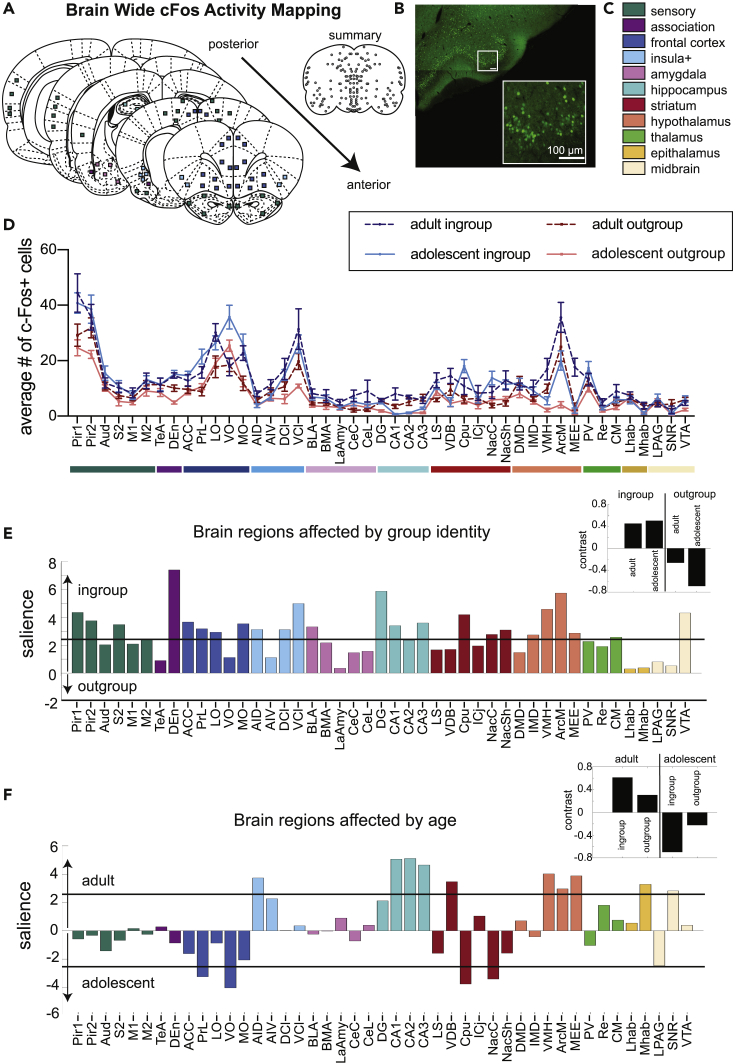
Neural activity associated with the helping behavior test The Brain-wide pattern of neural activity was determined by age and group identity. (A) Diagram of brain regions sampled for c-Fos expression. (B) A representative image of c-Fos signal sampled in the piriform cortex. (C) Legend of brain region categories coded by color. (D) Number of c-Fos + cells per region (mean ± SEM). Significant latent variables reveal that group identity (E) and age (F) determine neural activity patterns. The salience represents the z-score of boot-strapping tests, with regions crossing the black threshold lines significantly (p < 0.01) contributing to the contrast depicted in the inset (black bars). The directionality of the bars is congruent with the contrast graphs, as demonstrated by the arrows along the y axis. All regions were more active for ingroup than outgroup members, but several regions (e.g., VO) were more active for adolescent rats than adult rats. See also Figure S1 for detailed scatterplots of each region.

To map brain-wide activation associated with the HBT across development, the immediate early-gene c-Fos was quantified as an index of neural activity across a large number of brain regions. This strategy has previously proved useful for understanding whole-brain neural activity underlying complex behaviors including fear learning and other social behaviors (Oliveira, 2013; Rogers-Carter et al., 2018; Vetere et al., 2017; Wheeler et al., 2013; Ben-Ami Bartal et al., 2021). c-Fos was measured immediately following the final testing session during which the restrainer was latched shut, reflecting neural activity of rats in the presence of a trapped ingroup or outgroup member (n = 84 sampled brain regions per rat, Figures 3A–3D, see detailed STAR methods in (Ben-Ami Bartal et al., 2021). Thus, on this final day, all rats in all HBT conditions were in the presence of a trapped rat for the session’s entire duration and had an objectively similar experience, despite their different history of opening.

Two overarching patterns of neural activity were identified for the four HBT conditions using multivariate task partial least-square (PLS) analysis as previously described (Wheeler et al., 2013; Vetere et al., 2017; Ben-Ami Bartal et al., 2021). This analysis aims to identify patterns associated with each condition by maximizing the contrast between the tasks in a non-biased way. Two significant latent variables (LVs) emerged from data based on these four conditions, each one associated with a different pattern of neural activity, identified by permutation bootstrapping tests. One LV was associated with group identity (ingroup vs. outgroup, LV1, p < 0.001, Figure 3E), and the other was associated with age (adolescent vs. adult, LV2, p < 0.001, Figure 3F).

For both adolescent and adult rats, a distinct pattern of c-Fos activity emerged that was dependent on group identity. Specifically, exposure to a trapped ingroup member led to increased neural activity in a large number of brain regions, including in key regions previously observed to be uniquely active for ingroup relative to outgroup members in adults, such as the nucleus accumbens (Nac), lateral septum (LS), prelimbic cortex (PrL), and medial orbitofrontal cortex (MO) (Ben-Ami Bartal et al., 2021). Thus, regardless of age, the presence of trapped ingroup members recruits broad neural activity, indicating this is a more salient stimulus than a trapped outgroup member.

Although the first LV suggests most neural activity can be explained by group identity, the second LV emphasized overarching effects of age on neural activity, regardless of group identity. This LV can thus point to brain regions that are affected by development rather than social context; it revealed that adolescents displayed significantly reduced activity in the hippocampus, hypothalamus, and dorsal anterior insula, as well as increased frontal activity compared to adults. Effects in the striatum were mixed, with reduced activity in the vertical limb of the diagonal band of Broca (VDB) and increased activity in the caudate putamen (Cpu) and nucleus accumbens shell (NacSh) for adolescents compared to adults (Figure 3F).

**Figure 4:**
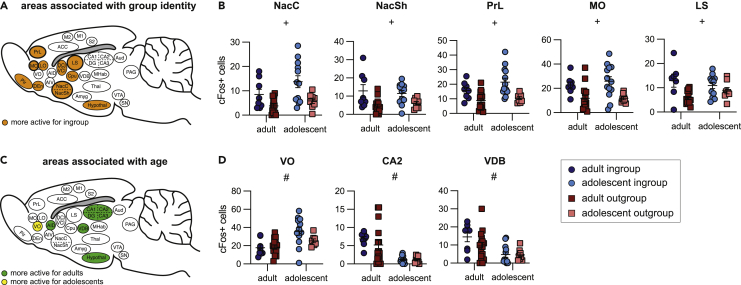
Neural activity of each condition, with main effects of group identity and age Brain activity associated with group identity. (A–B) and age (C–D) assessed by 2-way ANOVAs. (A) Brain diagram of all regions associated with group identity. All regions shown were more active for the ingroup than outgroup. (B) Scatterplots of five regions previously found to be uniquely active for adult ingroup compared to outgroup rats. Each region shows a main effect of group identity and adolescents display similar patterns as adults. (C) Brain diagram of all regions associated with age. Colored regions on the diagram represent areas more active for adults (green) or for adolescents (yellow). (D) Scatterplots of five of the seven brain regions that uniquely had a main effect of age but not group identity (not shown: AIV and CA3). All scatterplots can be found in Figure S1. Table S1 includes a detailed table for all regions. 2-way ANOVA results: + main effect of group identity, # main effect of age, § significant interaction between age and group identity. Error bars represent S.E.M.

To gain a better understanding of the interactions between group identity and age for each brain region, two-way ANOVAs with Bonferroni-corrected post hoc tests were used to compare cFos + cell numbers across the four HBT conditions (Figure 4, Table S1). A false discovery rate (FDR) method was further used to correct for multiple comparisons (Benjamini et al., 2006). As expected from the LVs described earlier, some regions showed group-identity effects, others showed age effects, and some regions were impacted by both. Based on the significant LVs, results are presented for group identity (Figures 4A and 4B) and age (Figures 4C and 4D) separately; a full display of scatterplots is available in Figure S1.

First, we focused on regions of interest previously found to be more active for adult ingroup than outgroup members, based on (Ben-Ami Bartal et al., 2021), (Figures 4A and 4B). These regions, the nucleus accumbens core (NacC), NacSh PrL, MO, and LS, all displayed main effects of condition (Table S1). Similar to adults, adolescents tested with ingroup members demonstrated increased c-Fos + cell numbers in the NacC, PrL, and MO (Bonferroni, p < 0.05) relative to adolescents tested with outgroup members. In contrast, c-Fos numbers within the NacSh and LS were not significantly different across adolescent groups despite a main effect for group identity, pointing to developed sensitivity to group-identity in these regions. In addition, all five of these regions did not show a main effect of age, further indication that group identity rather than age drives these observed patterns of c-Fos activity. Conversely, to highlight age-associated effects, we examined regions contributing to the age LV but not the group LV in the PLS analysis, meaning these regions did not pass the significance threshold in the group identity salience plot (Figures 4C and 4D). We found that the VDB and CA2 of the hippocampus were more active for adults, whereas the ventral orbitofrontal cortex (VO) was more active for adolescents (Figure 4D). Thus, developmentally dependent increases in activity in these regions could indirectly explain the social selectivity in helping behavior observed in adults.

### Increased amygdala connectivity for adolescent non-openers

**Figure 5:**
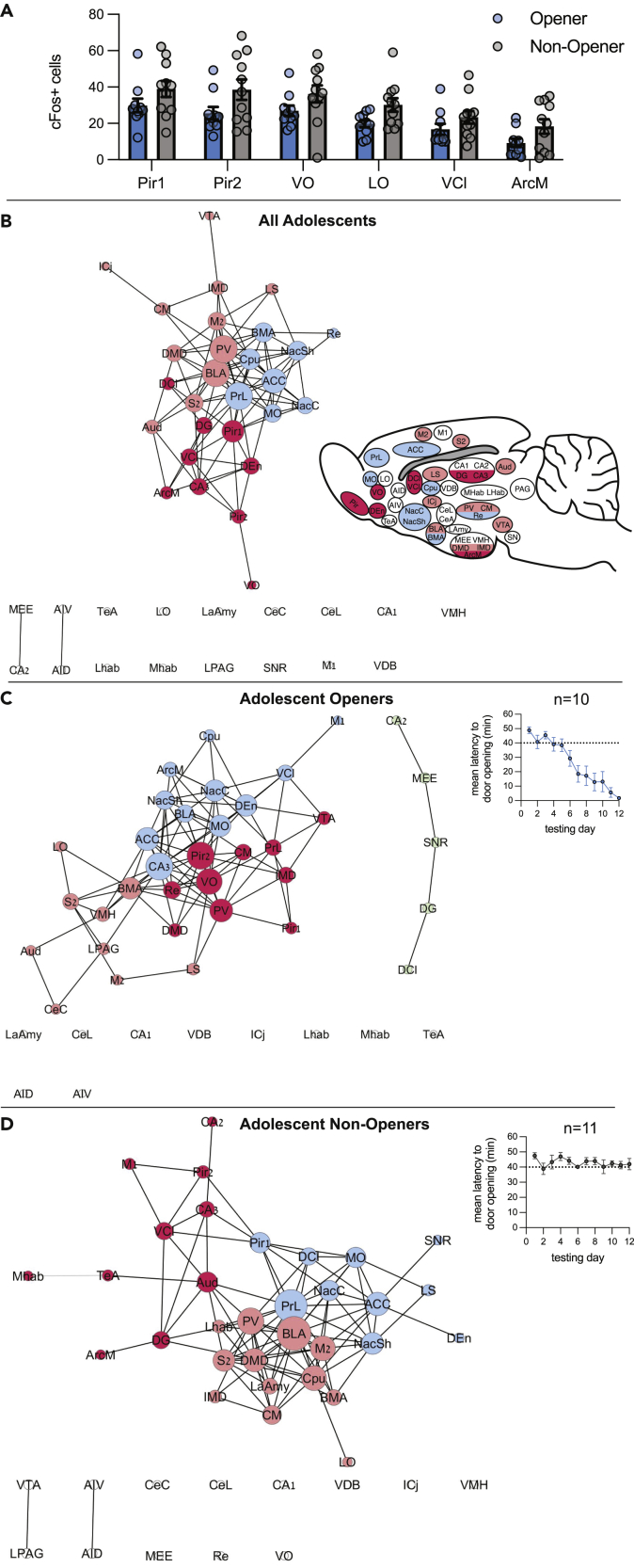
Different neural patterns for opener and non-opener adolescent rats Different neural patterns for opener and non-opener adolescent rats. (A) Brain regions with significantly higher levels of c-Fos for adolescent openers vs. non-openers are presented. Error bars represent S.E.M. (B–D) Network maps for adolescents tested in the HBT. (B) Network map for all adolescents, including rats in both the ingroup and outgroup condition. Inset: brain diagram colored by network clusters. (C) Network map for adolescent rats that became consistent openers. Inset: mean latency to door opening. (D) Network map for adolescent rats that did not consistently open across testing days. Inset: mean latency to door opening. See also Figure S5 for correlation matrices and central hubs of this data.

Although adolescents in general were motivated to release the trapped rat, not all of them became successful helpers; these rats were classified as “non-openers” (see STAR methods). When c-Fos levels were compared between openers and non-openers, a significant interaction emerged between opening and brain region (ANOVA, p < 0.05), stemming from significantly more activity for non-openers in the ventral and lateral orbitofrontal cortex (OFC), piriform cortex, ventral claustrum (VCl), and medial arcuate hypothalamus (ArcM) (Bonferroni, p < 0.05, Figure 5A).

To gain insight into the way different adolescent brain regions interact during the HBT, network graph theory was used to generate functional connectivity maps based on c-Fos quantification. The networks present the top 10% correlated regions, based on a Pearson’s pairwise correlation matrix (Figure S2) and clustered using a Louvain algorithm as previously reported in detail (Ben-Ami Bartal et al., 2021). Note that this analysis highlights areas that are highly correlated with other brain regions; it does not describe overall activity levels. Using this method, a network map for all adolescent rats revealed 3 central clusters. Brain regions such as the PrL, MO, and NAc, areas previously observed to be uniquely active in adult rats tested with cagemates, were also highly connected in one cluster of the network, alongside regions associated with empathy (Decety et al., 2016) such as the anterior cingulate cortex (ACC), suggesting that this network may be involved in the motivation to help in adolescents as well as in adults (Figure 5B). Interestingly, mirroring the PLS and ANOVA findings, both the insula and the CA2 were not part of the adolescent network, and neither were areas associated with aversive responses (lateral and central amygdala, habenula, and others), indicating that these brain regions are not central to the adolescent response to a trapped cagemate.

We next examined the brain-wide patterns of functional connectivity by graphing the network maps for adolescent openers and non-openers. This analysis revealed that the main “motivational” cluster described before was largely conserved in both openers and non-opener networks, including connectivity between the MO, ACC, and Nac (Figures 5C and 5D). However, for non-openers, a cluster containing amygdala regions emerged, including the basomedial, basolateral, and lateral amygdala (BMA, BLA, and LaAmy) and the habenula, indicating, in line with recent literature (Wu et al., 2021) that connectivity in the amygdala may be detrimental to helping (Figure 5D). Together, these findings demonstrate that common brain networks involved in reward and motivation were active in all adolescent rats regardless of door opening behavior.

## Discussion

This study aimed to examine the neural development of social bias for prosocial behavior in adolescent rats. We found that in contrast with adults, adolescent rats did not show an ingroup bias and instead helped trapped outgroup members, indicating that ingroup bias in rats emerges along development. One way to interpret the generalized helping in adolescent rats is a lack of sensitivity to group identity information because of later development of the neural circuits described before. However, the differences in movement patterns and social interactions provide behavioral evidence that adolescent rats do in fact distinguish between these social groups. An alternative explanation is that adolescents extend prosocial motivation to outgroup members perhaps because of increased salience of social stimuli compared to adults or a lack of threat arousal toward these adolescent outgroup conspecifics. In support of this explanation, we found increased exploratory interactions in the adolescent outgroup condition, compared to adults tested with outgroup members. As more affiliative interactions such as play fighting was observed for adolescent ingroup members, it is also possible that a different affective response was associated with each condition. Importantly, in contrast with adults, adolescents demonstrated prosocial motivation toward the trapped outgroup members, not indifference or threat arousal as observed for the adults. Although it is impossible to determine from these experiments if social reward, social investigation, or empathic arousal was the main motivator for helping, the difference between adults and adolescents toward outgroup members is striking. In addition, though social reward is experienced in this paradigm, the restraint stress in the HBT is by its very nature a stress-inducing task and it induces a stress response in both the free and trapped rat (Ben-Ami Bartal et al., 2016), including secretion of corticosterone, defecation, and freezing behavior (Glavin et al., 1994; Servatius et al., 2000). Thus, it is important to consider the distress of the trapped rat while interpreting these results. Besides, the trapped rats may respond differently to a free outgroup member than they would to a free ingroup member, as they, like the free rats, have never had exposure to the outgroup strain before testing. As it has previously been shown that signals of need are an important part of prosocial behavior (Marquez et al., 2015), both the distress of the trapped rat and the communication between the free and trapped rat are important topics that require further investigation.

An alternative explanation for the observed behavioral findings could stem from differing types of communication between rats across these conditions. Although the "free" rats in the ingroup condition were housed with the trapped cagemate, free rats in the outgroup condition were housed with another free rat. Thus, rats in the ingroup condition may have been receiving cues (e.g., distress signals) from their cagemate that could potentially influence their behavior during the subsequent sessions. However, this is unlikely to explain the behavioral difference between the adult and adolescent outgroup conditions, as these had the same housing experience with another free rat. Another difference between conditions is the level of familiarity with the trapped rat. Although the ingroup rats were familiar with the trapped rat (their cagemate), outgroup members lacked familiarity with the Long Evans strain as well as with the individual trapped rat. Thus, it is possible that familiarity with the trapped individual could have influenced the motivation to release that rat during subsequent sessions. However, we previously found that adult rats readily help unfamiliar others of their own strain as well as unfamiliar others of another strain, as long as they have familiarity with one member of that strain following two weeks of cohousing. Furthermore, fostered rats that were unexposed to their biological strain only helped strangers of their adoptive strain as adults (Ben-Ami Bartal et al., 2014). Together, these findings indicate that rats determine door-opening behavior by familiarity with the strain rather than individual familiarity. Future work should test for effects of familiarity in adolescent rats. However, here, the pattern of c-Fos activity in adolescents was similar to that observed in adults in the ingroup condition and was even somewhat enhanced. These findings render it unlikely that individual familiarity can sufficiently explain the observed helping behavior and lend support to the idea that social motivation is highly influenced by group categories in rats. Future experiments will focus on the neural differences in the response to familiar and unfamiliar ingroup members in the aim of identifying the generalization of social motivation to strangers observed in rats as is often the case in humans.

Here, adolescent rats tested in the HBT showed activation in a broadly dispersed neural network that responded preferentially to trapped ingroup members and was highly similar to that reported for empathy in adult rats (; Meyza et al., 2017) as well as in humans (Decety et al., 2016). This network includes regions in the sensory cortex, frontal cortex, ACC, anterior insula (AI), and reward and motivation areas like the claustrum, Cpu, Nac, hippocampus, and hypothalamus. A different pattern of neural activity for adolescents relative to adult rats may indirectly explain the lack of social selectivity in prosocial motivation in adolescents. Adolescent rats showed decreased activation in several regions compared to adults. Specifically, the CA2 and VDB were significantly less active for adolescents and were not modulated by group identity. Furthermore, the LS, an area identified as more active for adults tested with ingroup than outgroup members, was similarly active for adolescent rats in both conditions. This suggests that the discrimination that occurs in the LS for group membership in adulthood is not apparent during adolescence. Interestingly, in newborn rat pups, specific layers of the LS have been shown to be active in response to the pup’s own mother and siblings, whereas other layers respond to another mother and her litter (Clemens et al., 2020). This suggests that at least some social identity information is represented in the LS in early life. It is possible then that the increased LS activity we see in adult ingroup vs. outgroup rats tested in the HBT represents a separate subpopulation that is specifically important for prosocial responding.

In general, our results join with findings from other research groups showing neural sensitivity to social information across multiple brain regions, including in sensory and motivational networks. For example, in humans, responses in primary somatosensory cortex to touch are influenced by the perceived sex of the touching individual (Gazzola et al., 2012), and in mice, auditory cortex responses to pup alarm calls depend on previous exposure to maternal care (Carcea et al., 2021). In the olfactory system, sex-specific responses have been demonstrated along the vomeronasal pathway (Li and Dulac, 2018), and selective cohorts of mitral cells in the olfactory bulb respond to male urine compounds (Lin et al., 2005). Here, we similarly find that many sensory regions were differentially activated depending on group identity. Motivational regions can also be modulated by social identity information. The LS, a region involved in prosocial behavior in humans (Singer and Lamm, 2009) and rodents (Ben-Ami Bartal et al., 2021), participates in social recognition (Lukas et al., 2011) and is sensitive to kinship information (Clemens et al., 2020), as mentioned before. In addition, neuronal ensembles in the medial amygdala show sensitivity to conspecific sex in adult but not adolescent mice (Yao et al., 2017; Bergan et al., 2014). The mouse hypothalamus integrates information about conspecific sex and reproductive state (Kohl et al., 2018), and the dmPFC has been shown to respond to conspecific sex in mice (Kingsbury et al., 2020) and primates (Baez-Mendoza et al., 2021). Here, many of these same motivational regions were sensitive to group identity information, perhaps indicating that they contributed to the observed behavior.

However, the source of social identity information as well as the directionality of information flow between these regions is unclear. Thus, selective responding based on social group could be represented in these regions because of downstream incorporation of social identity, which drives differential affective and motivational responses. Of particular interest here, the CA2 is a hippocampal region key to social mapping Leroy et al., 2021 that participates in discriminating between familiar and unfamiliar others (Raam et al., 2017). Such socially sensitive regions may be part of a neural circuit that connects information about social identity with motivated behavior. In particular, both VDB — a cholinergic basal forebrain region inhibiting magnocellular cells — and the LS are structurally connected to the hippocampus and may be modulated by the CA2 (Hitti and Siegelbaum, 2014). Reduced hippocampal activation in adolescents may indicate a role for this region in the ingroup bias that emerges in adulthood. Here, we examined functional networks in all adolescent rats and found that both the CA2 and insula regions were not functionally connected to the main network, reinforcing the finding that these regions are not centrally involved in the HBT task before adulthood. Together, these findings support the hypothesis that CA2 becomes both more active and functionally connected to the rest of the brain in adulthood and participates in suppression of helping behavior toward nonaffiliated others.

The OFC also emerged as an area of interest in this study. We previously found increased activity in the OFC for adult rats tested with both ingroup and outgroup members compared to baseline, with MO being significantly more active for the ingroup condition (Ben-Ami Bartal et al., 2021). Here we found a similar trend, where MO and LO were significantly more active for adolescent ingroup members. Conversely, activity in the VO was not modulated by group identity, but it was impacted by age; the VO was the only region that was significantly more active for adolescents than adults. Interestingly, the VO was even more active in adolescent non-openers. As the OFC participates in processing rewards and evaluating outcomes (Simon and Moghaddam, 2015), its specific modulation by group identity and success at helping may reflect involvement of the OFC in placing a value on the outcome of the trapped rat.

### Limitations of the study

The current study faces several methodological limitations. First, owing to a limited number of arenas that could be run simultaneously, adolescent animals were run through the HBT following experiments with adults. We sought to minimize differences in nonexperimental parameters such as ensuring continuity in experimenters conducting behavior, and thus, we do not anticipate the staggered timelines that influenced our results. However, future work should conduct a side-by-side comparison of the HBT in adults and adolescents. Second, there are limitations with using c-Fos staining; these have been extensively described in prior work (Ben-Ami Bartal et al., 2021). Critically, c-Fos staining results in low temporal resolution, and thus, future work can expand upon the current study by using technology such as fiber photometry or activity-targeted viral vectors to assess neural activity in adolescent rats undergoing the HBT. Higher temporal resolution will provide insight into neural activity during learning across the task, during door opening behavior, and during subsequent social interactions, which we found differed according to group identity in adolescents. Here, our methodology using whole brain c-Fos adds to the growing validation of this type of unbiased approach in looking at brain activity in complex behaviors (Turkheimer et al., 2021). Our data suggest several key brain regions that may be responsible for helping behavior in adolescent rats. Future work will be able to expand on our findings to target specific regions and circuits, with the goal of artificially manipulating prosocial motivation across development. It is also important to note that the behavioral and neural findings here are from male rats. We are currently collecting data from both adult and adolescent female rats; how sex interacts with prosocial motivation will be critical to provide a more complete understanding of factors contributing to biases in helping behavior. Lastly, we observed individual variability in helping behavior across the four conditions. These differences, along with a different number of subjects in each condition, weaken our ability to attribute a social motivation at the group level. Future experiments should aim to increase the number of subjects in these conditions and to explore individual variability further.

Our finding that adolescents help nonaffiliated others opens up new areas for future investigation. Behaviorally, one hypothesis is that exposure to an outgroup member early in development may be sufficient to reduce biases in prosocial behavior. It will be worth exploring the bounds of this hypothesis; for example, is there a developmental window in which social context contributes to bias? Besides, would a brief exposure of adolescent SD rats to LE strangers drive prosocial helping when tested as adults? Alternatively, adolescent rats may be driven to open for outgroup members because of social novelty or a desire for social investigation as suggested from our social interaction data. Future studies will be able to directly address these hypotheses through manipulation of the early social environment and through manipulation of social interaction following door-opening. Interestingly, here we found that adolescent rats were less successful at helping trapped ingroup members compared to outgroup members. Though this could suggest reduced motivation to release trapped cagemates, both movement data and increased neural activity suggest they were highly motivated to do so. Future studies should explore this finding further. For example, it is possible that trapped cagemates were less stressed because of social buffering. Alternatively, on the other end of the spectrum, free adolescent rats may have experienced high levels of emotional contagion to their trapped cagemate, inhibiting focused and directed behavior. Future studies could analyze corticosterone secretions in adolescent ingroup and outgroup conditions to assess these possibilities and to assess the motivational state of the adolescent ingroup condition. Lastly, future work should test adolescent rats in the HBT with same-strain unfamiliar peers to distinguish between strain vs familiarity.

Future work should also expand upon the neural data of the current study. In particular, we observed increased activity in the OFC, VCl, and ArcM for non-opener compared to opener adolescents. One possibility is that this may stem from an increased motivation in the non-openers, if indeed they were motivated to release the trapped rat. Further experiments will be needed to understand whether activity in these regions inhibits helping or whether it reflects continued motivation. In addition, on a neural level, our findings suggest there may be a developmental trajectory of circuits that are not yet active in adolescents, including in the hippocampus and insula. Future work can test exactly when in development these brain regions become engaged in the larger network. In addition, future work could test the hypothesis that activation of hippocampal and/or insula regions are responsible for inhibition of helping outgroup members.

In conclusion, this study sheds light on the developmental basis of prosocial motivation and in-group bias. We demonstrate for the first time that adolescent rats are capable of helping behavior and help distressed others regardless of group identity. Further, we provide a window into the neural circuits associated with helping across development. Adolescent rats show a different pattern of neural activity during the HBT than adults; these differences may indirectly explain the lack of ingroup bias in adolescent rats. In particular, our results put a spotlight on the hippocampus and its role in group categorization and suggest that in adults, CA2 activity may inhibit indiscriminate helping behavior. Overall, this study provides evidence for a developmental basis of prosocial helping across mammalian species and highlights a distinct neural response to the distress of affiliated others depending on age and group identity.

## STAR★Methods

### Key resources table

**Table:** 

REAGENT or RESOURCE	SOURCE	IDENTIFIER
**Antibodies**		

Rabbit anti-cFos primary antibody	Millipore Sigma	Millipore: ABE457; RRID: AB_2631318
Donkey anti-rabbit IgG Alexa Fluor 488 secondary antibody	Jackson ImmunoResearch Labs	Cat#: 711-545-152; RRID: AB_2313584

**Deposited data**		

https://osf.io/6b2qc/	N/A	This paper

**Experimental models: Organisms/strains**		

Sprague-Dawley Rat	Charles River Labs	Charles River 001; RRID: RGD_10395233
Long-Evans Rat	Envigo	Envigo: HsdBlue:LE; RRID: RGD_5508398

**Software and algorithms**		

MATLAB	Mathworks (https://www.mathworks.com)	RRID: SCR_001622
SPSS	IBM	RRID:SCR_019096
Zeiss ZEN 2 (Blue)	Zeiss	RRID: SCR_013672
Fiji ImageJ	NIH (https://imagej.net/Fiji/Downloads); (Schneider et al., 2012)	RRID: SCR_002285
Behavioral Observation Research Interactive Software Project (BORIS)	https://edspace.american.edu/openbehavior/project/boris/	RRID:SCR_021434
GraphPad Prism	http://www.graphpad.com/	RRID: SCR_002798

### Resource availability

#### Lead contact

Further information and requests for resources and reagents should be directed to and will be fulfilled by the lead contact, Inbal Ben-Ami Bartal (inbalbe@tauex.tau.ac.il).

#### Materials availability

This study did not generate any new reagents or animal lines.

### Experimental model and subject details

#### Animals

Rat studies were performed in accordance with protocols approved by the Institutional Animal Care and Use Committee at the University of California, Berkeley. Rats were socially housed in cages of two same sex individuals, in a temperature (22–24C) and humidity controlled (55% relative humidity) animal facility, on a 12:12 light:dark cycle (lights on at 07:00). Food and Water was provided *ad libitum*. All testing was done in the rat’s light cycle. In total, 45 male rats were tested as the free rat across all experiments; brains from these animals were collected for subsequent processing (‘adolescent ingroup’, n = 13; adolescent outgroup’ n = 8; adult ingroup’ n = 8; ‘adult outgroup’, n = 16). 45 additional male rats were used as trapped rats across all experiments. For experiments with adults, male Sprague-Dawley rats (age postnatal day (p) 60-p90 days) were used as the free & trapped ingroup rats (Charles River, Portage, MI). Adult male Long-Evans rats were used as trapped outgroup rats (Envigo, CA). For experiments with adolescents, Sprague-Dawley (Charles River) rats were born in-house at UC Berkeley. Six different breeding pairs were set up for in-house breeding. Litters ranged in size from 8–16 pups per litter, with an average of 12 pups per litter. Animals were separated by sex and weaned at p21, then were housed in pairs one week later at p28. Male rats from 6 different litters were used for these experiments. Male Long-Evans rats (p28) housed in pairs were purchased from Charles River, as our Long-Evans breeders did not get pregnant as expected. All rats that were ordered were allowed a minimum of 5 days to acclimate to the facility prior to beginning testing. Trapped and free rats were of the same sex and age. Sprague Dawley animals were assigned to one of two experimental groups: they were either tested with cagemates (ingroup) or with Long-Evans strangers (outgroup). In the outgroup condition, the trapped rats as well as the free rat had never been exposed to a rat of the other strain before the first day of testing. The only exposure was such that was afforded by the 1h testing sessions for the two weeks of behavioral testing, which was previously shown to be insufficient to lead to door-opening (Ben-Ami Bartal et al., 2014). All rats were pair-housed. Rats in the ingroup conditions were housed with the trapped cagemate they were encountering during the sessions of the helping behavior test. Rats in the outgroup condition were housed with a cagemate who was also tested as a free rat in the helping behavior test with a stranger of the other strain. As such, adolescents and adults within social condition experienced identical housing conditions.

### Method details

#### Helping behavior test (HBT)

The helping behavior test (HBT) was performed as described previously (Ben-Ami Bartal et al., 2011). Briefly, animals underwent five days of handling prior to starting the HBT. In addition to handling, on days 2–4, animals were given 30-min habituation sessions where they were placed in an empty arena (50 × 50cm) with their cagemate. On day 5, animals underwent a 15-min open field task in the same arenas, one animal at a time. For the HBT, rats were tested in 60-min sessions over a 12-day period. On each day, rats were placed into arenas with either a trapped Sprague-Dawley rat (‘ingroup’) or Long-Evans rat (‘outgroup’) inside a plexiglass restrainer (25 x 8.75 x 7.5cm) located at the center of the arena (Harvard Apparatus, Holliston MA). As previously described, the door to the arena could only be opened by the free rat on the outside, either by pulling the door from the top or side or by pushing the door open with its snout (Ben-Ami Bartal et al., 2011). As in prior work, if the free rat did not open the restrainer after 40 min, the door was opened half-way by the experimenter. Both rats remained in the arena for the full hour. If the free rat opened the door before the half-way opening it was counted as a door-opening. After the initial 12 days, following a delay of typically one week, rats underwent three more test days. On the last day of testing, the restrainer was latched shut throughout the 60-min session and rats were perfused within 30 min of completing behavioral testing. Restrainers were latched on the final day such that rats in all HBT conditions were in the presence of a trapped rat for the session’s entire duration and had an objectively similar experience on the final test day, despite their different history of opening. ‘Openers’ were defined as rats who opened the restrainer on at least two of the last three sessions (prior to the final day where the restrainers were latched shut). Sessions were video recorded with a CCD color camera (KT&C Co, Seoul, Korea) connected to a video card (Geovision, Irvine, CA) that linked to a PC. Movement data were analyzed using Ethovision video tracking software (Noldus Information Technology, Inc. Leesburg, VA). All adolescents began the first day of restrainer testing at approximately p32, while adults began the HBT between ages p60-p90.

#### Social interaction scoring

Five minutes of behavior was analyzed immediately upon release using BORIS software (see Key resources table). For rats that did not open the restrainer after 40 min, these interactions occurred in the final 20 min of the session once the trapped rat released himself. Two major categories of social behavior were scored: 1) play fighting interactions, including pinning and wrestling, and 2) non-play interactions, including nose to nose and nose to body touching and anogenital sniffs. Several videos could not be scored to due to video encoding and export errors.

#### Immunohistochemistry

On the last day of testing, animals were sacrificed within 90 min from the beginning of the session, at the peak expression of the early immediate gene product c-Fos. Rats were transcardially perfused with 0.9% saline and freshly made 4% paraformaldehyde in phosphate buffered saline (PBS). Brains were then sunk in 30% sucrose as a cryoprotectant and frozen at −80 °C. They were later sliced at 40 μm and stained for c-Fos, as has been previously reported(Ben-Ami Bartal et al., 2021). Sections were washed with 0.1M tris-buffered saline (TBS; 3 × 5′), incubated in 3% normal donkey serum (NDS) in 0.3% Triton X-100 in TBS (TxTBS), then transferred to rabbit anti-*c*-Fos antiserum (ABE457; Millipore, 1:1000; 1% NDS; 0.3% TxTBS) overnight. Sections were then washed in 0.1M TBS (3 × 5′), and incubated in Alexa Fluor 488-conjugated donkey anti-rabbit antiserum (AF488; Jackson, 1:500; 1% NDS; 0.3% TxTBS). Sections were then briefly washed in 0.1M TBS again (3 × 5′). Sections were further stained in DAPI (1:40,000), then washed for an additional 15 min (3 × 5′). Lastly, all slides were coverslipped with DABCO, dried overnight and stored at 4 °C until imaged.

Immunostained tissue was imaged at 10× using a wide field fluorescence microscope (Zeiss AxioScan) and was processed in Zen software. Regions of interest (250 × 250 μ m squares) were placed across the whole brain, as described in(Ben-Ami Bartal et al., 2021). A custom written script in ImageJ V2.0.0 (National Institute of Health, Bethesda, MD) was used to quantify immunoreactive nuclei, followed by manual checks and counting by multiple individuals who were blind to condition; consistency for counts across individuals was verified by a subset of samples. The threshold for detection of positive nuclei was set at a consistent level for each brain region, and only targets within the size range of 25–125 mm^2^ in area were counted as cells. Manual verification was targeted at identifying gross errors in the ImageJ scripts. For instance, in some cases the script falsely identified >100 cells within the counting square; this usually occurred when there was high background staining. This type of error occurred in ∼15% of the samples, which were then manually corrected. 39 values for cell counts were removed from the dataset as outliers. Outliers were defined as those that were more than two standard deviations higher or lower than the group mean and further fell outside of the observed range for all conditions.

### Quantification and statistical analyses

Statistical details can be found within the Results section. In all written description and figures, n represents the number of animals in each condition. All means are reported as mean ± SEM Statistical analyses described below were performed using MATLAB, SPSS, and Graphpad Prism.

#### Task partial least square (PLS) analysis

Task PLS is a multivariate statistical technique that has been used to identify optimal patterns of activity that differentiate conditions (Mcintosh et al., 1996; Mcintosh, 1999). Task PLS is used in the analysis of brain region activity to describe the relationship between experimental conditions and correlated activity. PLS identifies similarities and differences between groups by locating regions where activation varies with the experimental condition. Through singular value decomposition, PLS produces a set of mutually orthogonal latent variable (LV) pairs. One element of the LV depicts the contrast, which reflects a commonality or difference between conditions. The other element of the LV, the brain region salience, identifies brain regions that show the activation profile across tasks, indicating which brain areas are maximally expressed in a particular LV.

Statistical assessment of PLS was performed by using permutation testing for latent variables (LVs) and bootstrap estimation of SEstandard error for the brain region saliences. For the LV, significance was assessed by permutation testing: resampling without replacement by shuffling the test condition. Following each resampling, the PLS was recalculated. This was done 500 times in order to determine whether the effects represented in a given LV were significantly different than random noise. For brain region salience, reliability was assessed using bootstrap estimation of standard error. Bootstrap tests were performed by resampling 500 times with replacement, while keeping the subjects assigned to their conditions. This reflects the reliability of the contribution of that brain region to the LV. Brain regions with a bootstrap ratio greater than 2.55 (roughly corresponding to a confidence interval of 99%) were considered as reliably contributing to the pattern. Missing values were interpolated by the average for the test condition. An advantage to using this approach over univariate methods is that no corrections for multiple comparisons are necessary because the brain region saliences are calculated on all brain regions in a single mathematical step.

#### Network analysis

Network graphs were generated by first obtaining a correlation matrix of c-Fos activity between all brain regions (using pairwise Pearson correlation coefficients). The top 10% of correlations were presented in a graphic form. This cutoff threshold of 10% was determined based on scale-free network characteristics in prior work (Ben-Ami Bartal et al., 2021) and used here for comparability. Correlation values higher than the cutoff were set to one and the corresponding brain regions greater than 1 were considered connected to the network. See (Ben-Ami Bartal et al., 2021) for detailed STAR methods.

#### Other statistical tests

In addition to the PLS analysis described above, two-way ANOVAs were conducted on the c-Fos data to compare the four HBT conditions and to assess main effects of age (adult vs. adolescent) and group identity (ingroup vs outgroup). 2-way ANOVAs were also used to compare the pattern of animals’ movements during testing. Bonferroni post hoc corrections were used following all ANOVAs. A False Discovery Rate method was further used to correct for testing multiple regions, specifically the two-stage linear step-up procedure of Benjamini, Krieger and Yekutieli (Benjamini et al., 2006). Changes across days to helping behavior, including % door-opening and latency to door-opening, were examined using the non-parametric Cochran’s Q test and Friedman test respectively.

## Data Availability

All data have been uploaded in the following Open Science Framework depository (https://osf.io/6b2qc/) which is publicly available as of the date of this publication. DOIs are listed in the key resources table. This paper does not report original code. Any additional information required to reanalyze the data reported in this paper is available from the lead contact upon request.
