# Supplementary material for: Neural activation associated with outgroup helping in adolescent rats
Source: iScience. 2022 May 16;25(6):104412. doi: 10.1016/j.isci.2022.104412 (PMC9160754; doi:10.1016/j.isci.2022.104412)
Supplement: Document S1. Figures S1 and S2 and Table S1 [file mmc1.pdf]

iScience, Volume *25*

Supplemental information

Neural activation associated with outgroup helping in adolescent rats

Jocelyn M. Breton, Jordan S. Eisner, Vaidehi S. Gandhi, Natalie Musick, Aileen Zhang, Kimberly L.P. Long, Olga S. Perloff, Kelsey Y. Hu, Chau M. Pham, Pooja Lalchandani, Matthew K. Barraza, Ben Kantor, Daniela Kaufer, and Inbal Ben-Ami Bartal

Supplemental Figures & Tables

Fig. S1. Individual c-Fos expression for all regions and conditions, related to Fig. 3

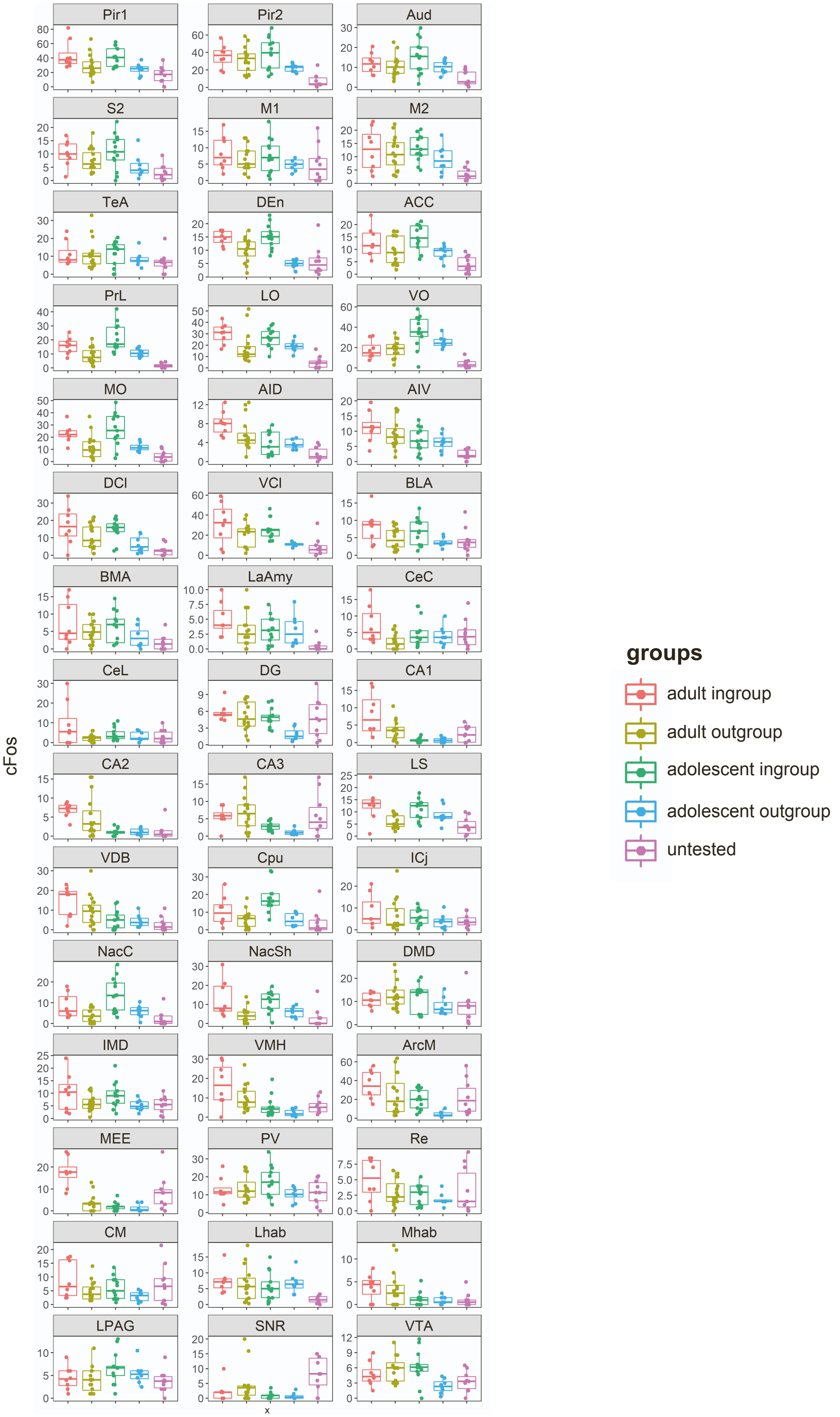

Fig S1. Individual c-Fos expression for all regions and conditions. Box plots of c-Fos data in all brain regions across all test groups. Center bars mark the median. Lower and upper edges correspond to the 25th and 75th percentiles. Descriptions of the brain region abbreviations can be found in Table S1. Data points are jittered along the x-axis to avoid overlaps. X: experimental groups; Y: c-Fos^{+} cell numbers.

Fig. S2. Central hubs for adolescent openers and non-openers, related to figure 5

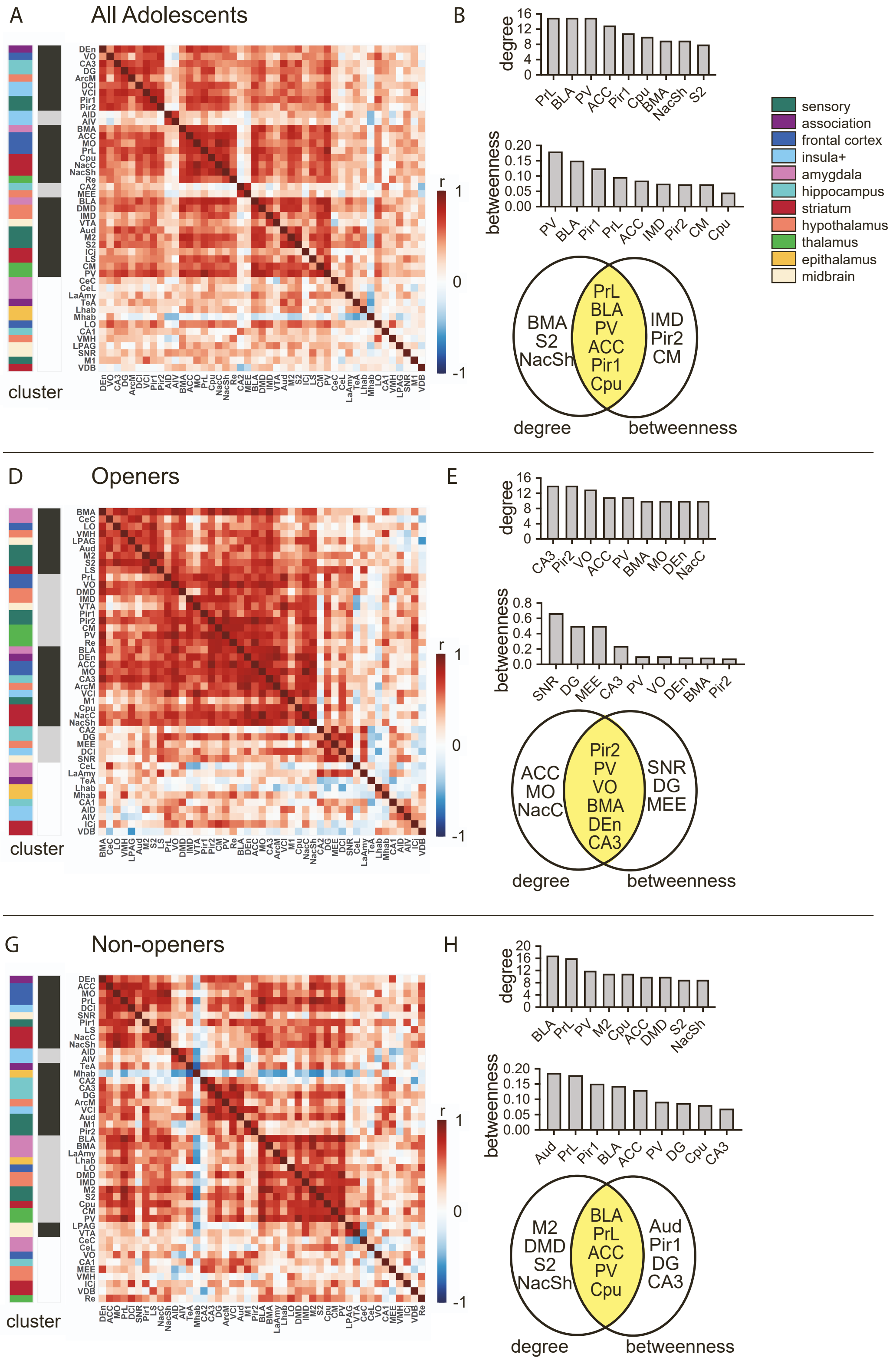

Fig S2. Pearson's correlations across all brain regions in adolescent rats tested in the HBT. Correlation matrices (A,C,E) and central hubs (B,D,F) for (A-B): All adolescent rats, (C-D): Adolescent openers, and (E-F) Adolescent non-openers. Colored bars on left indicate general brain region of each row (for color legend see Fig. 3) The grayscale bar denotes clusters determined via Louvain algorithm. The regions rated as top 20% for degree and connectivity were combined to identify central hubs.

Table S1. 2- way ANOVA results. Related to Figures 3-5

**Brain Region**	**Group Identity**	**Age**	**Interaction**
Pir1<br>Piriform c.	F (1, 40) = 11.63<br>** **q=0.0052**	F (1, 40) = 0.8078	F (1, 40) = 0.01069
Pir2<br>Piriform c.	F (1, 41) = 1.946	F (1, 41) = 0.5543	F (1, 41) = 5.036
Aud<br>Auditory c.	F (1, 39) = 2.566	F (1, 39) = 0.4879	F (1, 39) = 1.104
S2<br>Somatosensory c.	F (1, 39) = 5.648	F (1, 39) = 0.2088	F (1, 39) = 0.6472
M1<br>Motor c.	F (1, 38) = 2.608	F (1, 38) = 0.7417	F (1, 38) = 0.1015
M2<br>Motor c.	F (1, 42) = 2.593	F (1, 42) = 0.3220	F (1, 42) = 0.6461
TeA<br>Temporal assoc. c.	F (1, 38) = 0.4533	F (1, 38) = 0.3985	F (1, 38) = 0.5176
Den<br>Endopiriform	F (1, 40) = 37.94<br>** **q=0.0017**	F (1, 40) = 3.677	F (1, 40) = 5.219
ACC<br>Ant. Cingulate c.	F (1, 41) = 8.533	F (1, 41) = 0.03591	F (1, 41) = 0.8141
PrL<br>Prelimbic c.	F (1, 40) = 16.43<br>** **q=0.0017**	F (1, 40) = 2.943	F (1, 40) = 0.8072
LO<br>Lat. Orbitofrontal c.	F (1, 39) = 9.985<br>** **q=0.0086**	F (1, 39) = 0.3394	F (1, 39) = 0.2903
VO<br>Ventral orbitofrontal	F (1, 40) = 2.031	F (1, 40) = 13.20<br>****q=0.0031**	F (1, 40) = 2.549
MO<br>Med. Orbitorfrontal	F (1, 40) = 15.36<br>** **q=0.0021**	F (1, 40) = 0.2487	F (1, 40) = 0.2257
AID<br>Dorsal ant. Insula	F (1, 40) = 1.922	F (1, 40) = 13.55<br>** **q=0.0031**	F (1, 40) = 1.732
AIV<br>Ventral ant. insual	F (1, 41) = 1.470	F (1, 41) = 5.303	F (1, 41) = 0.7340
DCl<br>Dorsal claustrum	F (1, 41) = 12.14<br>** **q=0.0046**	F (1, 41) = 1.939	F (1, 41) = 0.4127
VCl<br>Ventral claustrum	F (1, 41) = 12.52<br>** **q=0.0043**	F (1, 41) = 3.991	F (1, 41) = 0.2145

BLA<br>Basolateral amygdala	F (1, 41) = 8.897	F (1, 41) = 0.8785	F (1, 41) = 0.03535
BMA<br>Basomedial amygdala	F (1, 39) = 3.372	F (1, 39) = 0.5021	F (1, 39) = 0.006811
LaAmy<br>Lateral amygdala	F (1, 40) = 0.7826	F (1, 40) = 0.9492	F (1, 40) = 1.604
CeC<br>Central amygdala	F (1, 40) = 6.181	F (1, 40) = 0.008356	F (1, 40) = 3.237
CeL<br>Central amygdala	F (1, 39) = 5.056	F (1, 39) = 1.232	F (1, 39) = 3.135
DG<br>Dentate gyrus	F (1, 40) = 8.677	F (1, 40) = 10.97<br>** **q=0.0071**	F (1, 40) = 3.633
CA1<br>hippocampus	F (1, 39) = 4.987	F (1, 39) = 25.58<br>*** **q=0.0009**	F (1, 39) = 4.846
CA2<br>Hippocampus	F (1, 40) = 0.6554	F (1, 40) = 20.93<br>*** **q=0.0009**	F (1, 40) = 0.6074
CA3<br>hippocampus	F (1, 38) = 0.09875	F (1, 38) = 14.97<br>** **q=0.0028**	F (1, 38) = 1.473
LS<br>Lateral septum	F (1, 39) = 11.24<br>** **q=0.0056**	F (1, 39) = 0.1116	F (1, 39) = 3.227
VDB<br>Vent. Diag. band of broca	F (1, 40) = 1.589	F (1, 40) = 13.02<br>** **q=0.0031**	F (1, 40) = 1.414
Cpu<br>Caudate putamen	F (1, 40) = 17.59<br>** **q=0.0017**	F (1, 40) = 2.950	F (1, 40) = 3.499
ICj<br>Islands of callaja	F (1, 39) = 1.060	F (1, 39) = 1.727	F (1, 39) = 1.178e-006
NAcC<br>Nuc. accumbens core	F (1, 40) = 12.52<br>** **q=0.0043**	F (1, 40) = 4.243	F (1, 40) = 0.8062
NAcSh<br>Nuc. accumbens shell	F (1, 40) = 14.73<br>** **q=0.0023**	F (1, 40) = 0.001683	F (1, 40) = 0.7023
DMD<br>Dorsomed. Hypothalamus	F (1, 40) = 0.8251	F (1, 40) = 1.978	F (1, 40) = 1.890
IMD<br>Int. mediodorsal hypothalamus	F (1, 41) = 7.645	F (1, 41) = 0.5442	F (1, 41) = 0.002939
VMH<br>Ventromedial hypothalamus	F (1, 41) = 6.212	F (1, 41) = 20.74<br>*** **q=0.0009**	F (1, 41) = 0.7902

ArcM<br>Med. Arcuate hypothalamus	F (1, 39) = 7.110	F (1, 39) = 13.41<br>**** q=0.0031**	F (1, 39) = 0.2829
MEE<br>Med. eminence	F (1, 31) = 18.03<br>**** q=0.0017**	F (1, 31) = 31.75<br>***** q=0.0009**	F (1, 31) = 14.40
PV<br>Paraventricular thalamic nu.	F (1, 39) = 2.405	F (1, 39) = 0.03630	F (1, 39) = 2.802
Re<br>Reunions	F (1, 40) = 5.066	F (1, 40) = 6.502	F (1, 40) = 0.7999
CM<br>Cent. Med thalamus	F (1, 41) = 7.399	F (1, 41) = 3.557	F (1, 41) = 0.1994
Lhab<br>Lateral habenula	F (1, 39) = 0.002036	F (1, 39) = 0.1812	F (1, 39) = 0.4681
Mhab<br>Medial habenula	F (1, 38) = 0.08974	F (1, 38) = 7.920	F (1, 38) = 0.001485
LPAG<br>Lateral periaqueductal gray	F (1, 35) = 0.5708	F (1, 35) = 2.982	F (1, 35) = 0.1695
SNR<br>Sabstantia nigra	F (1, 36) = 0.5972	F (1, 36) = 3.947	F (1, 36) = 0.8727
VTA<br>Ventral tegmental area	F (1, 36) = 1.913	F (1, 36) = 1.437	F (1, 36) = 7.306

Table S1. 2- way ANOVA results. Main effects of group identity, age, and/or interaction between the two. The F statistic is shown for each main effect. Multiple comparisons were corrected for using a False Discovery Rate (FDR) approach. Statistically significant discoveries and their FDR adjusted p-values (q-values) are shown in bold. *q<0.05, **q<0.01, ***q<0.001.
